# Analysis of Lane-Changing Decision-Making Behavior of Autonomous Vehicles Based on Molecular Dynamics

**DOI:** 10.3390/s22207748

**Published:** 2022-10-12

**Authors:** Dayi Qu, Kekun Zhang, Hui Song, Tao Wang, Shouchen Dai

**Affiliations:** School of Mechanical and Automotive Engineering, Qingdao University of Technology, Qingdao 266520, China

**Keywords:** intelligent transportation, autonomous vehicles, environmental perception, lane-changing decision-making behavior, molecular dynamics, interaction potential

## Abstract

Along with the rapid development of autonomous driving technology, autonomous vehicles are showing a trend of practicality and popularity. Autonomous vehicles perceive environmental information through sensors to provide a basis for the decision making of vehicles. Based on this, this paper investigates the lane-changing decision-making behavior of autonomous vehicles. First, the similarity between autonomous vehicles and moving molecules is sought based on a system-similarity analysis. The microscopic lane-changing behavior of vehicles is analyzed by the molecular-dynamics theory. Based on the objective quantification of the lane-changing intention, the interaction potential is further introduced to establish the molecular-dynamics lane-changing model. Second, the relationship between the lane-changing initial time and lane-changing completed time, and the dynamic influencing factors of the lane changing, were systematically analyzed to explore the influence of the microscopic lane-changing behavior on the macroscopic traffic flow. Finally, the SL2015 lane-changing model was compared with the molecular-dynamics lane-changing model using the SUMO platform. SUMO is an open-source and multimodal traffic experimental platform that can realize and evaluate traffic research. The results show that the speed fluctuation of autonomous vehicles under the molecular-dynamics lane-changing model was reduced by 15.45%, and the number of passed vehicles was increased by 5.93%, on average, which means that it has better safety, stability, and efficiency. The molecular-dynamics lane-changing model of autonomous vehicles takes into account the dynamic factors in the traffic scene, and it reasonably shows the characteristics of the lane-changing behavior for autonomous vehicles.

## 1. Introduction

With the promotion of an intelligent networking environment, autonomous driving technology has been developed, which alleviates the driving task of drivers to a certain extent, and thus makes travel safe and efficient [1]. Autonomous driving technology includes three key parts: perception, decision making, and control [2]. Among them, the decision-making part reflects the intelligence of autonomous vehicles. The lane-changing decision making is indispensable in autonomous driving decision making. Its main content is to judge the lane-changing feasibility and ensure the safety and efficiency of the lane-changing behavior [3]. In addition, lane-changing decision making plays a vital role in the smooth operation of vehicles. With the development of intelligent transportation technology, scholars have conducted relevant research on lane changing. According to different decision-making methods, the research on lane-changing decision making is mainly based on rules, artificial intelligence, and the utility function.

In terms of the rules, Gipps [4] divided the lane-changing process and established layered lane-changing rules, which laid the theoretical foundation for lane-changing decision making. Jula et al. [5] used kinematics to analyze lane-changing vehicles, studied the lane-changing conditions, and established lane-changing rules with a minimum longitudinal safety distance. Kanaris et al. [6] analyzed the lane-changing environment and established safety-evaluation rules based on the minimum lane-changing distance. Chen et al. [7] studied the decision-making behavior of vehicles under complex urban conditions and established reliable lane-changing rules based on safety and efficiency. Du et al. [8] established lane-changing rules according to the lateral acceleration, ensuring lane-changing safety and comfort. Zhao et al. [9] processed traffic-flow data, analyzed the speed, acceleration, and relative distance, and formulated lane-changing decision-making rules. Xu et al. [10] analyzed the trajectory data of vehicles and established lane-changing rules. Li et al. [11] considered spline theory in the lane-changing-trajectory function, established lane-changing rules in an expressway environment, and optimized the problem of the trajectory-curvature discontinuity.

In terms of artificial intelligence, lane-changing research is carried out around Bayesian theory and Markov decision theory. Schubert et al. [12] used a Bayesian algorithm to judge the current road conditions and built a lane-changing decision-making model based on Bayesian theory. Ulbrich et al. [13] used dynamic Bayesian theory and the traceless-variance-transformation method to evaluate the lane-changing state of vehicles. Qiu et al. [14] combined the Bayesian network and machine learning to establish a lane-changing model. Zhang et al. [15] used the Bayesian network to predict the lane-changing probability and evaluate the driving safety level. Gu et al. [16] studied the lane-changing problem in complex environments and optimized the lane-changing decision-making model based on the improved Bayesian algorithm. Kuge et al. [17] established a recognition model based on the hidden Markov model to effectively recognize lane-changing behavior. Hong et al. [18] combined the neural network and Markov decision to establish a lane-changing model, predicting the lane-changing frequency. Brechtel et al. [19] used the Markov decision method to optimize the lane-changing decision, and they analyzed the lane-changing characteristics through the weighted method.

In terms of the utility function, the decision making is established through utility thought. Furthermore, comfort, efficiency, and other factors are analyzed to make the lane-changing decision making reasonable. Nilsson et al. [20] added path and speed information to the utility function, and they ensured lane-changing comfort by controlling the acceleration of vehicles. Toledo et al. [21] studied the selection of target lanes and established a lane-changing model based on utility selection. Pan et al. [22] quantitatively described lane-changing behavior based on the linear utility function. Wang et al. [23] established a lane-changing utility motion model from the perspective of the lane-changing trajectory, which improved the defect of reaction delay. Feng et al. [24] established a lane-changing decision-making model based on binomial-random utility theory, and they applied it to free lane changing and forced lane changing. Zheng et al. [25] established a utility lane-changing model with the acceleration as the main variable to explore the microscopic lane-changing characteristics of vehicles. Gu et al. [26] analyzed the lane-changing process based on utility theory to maximize the lane-changing utility.

**Figure 1:**
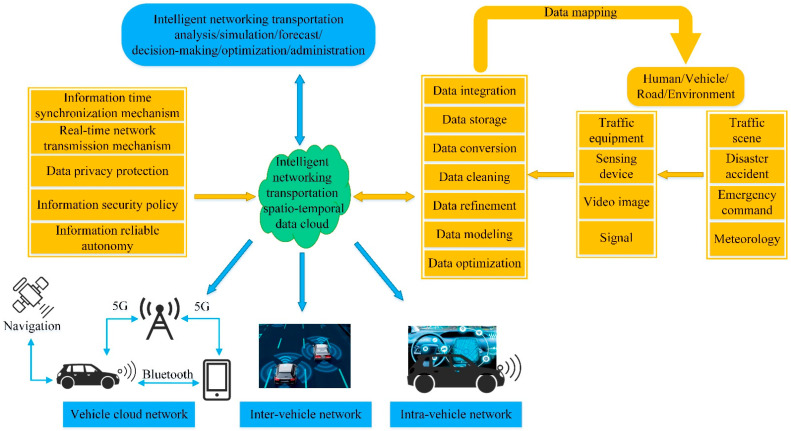
Intelligent networking transportation system.

Autonomous vehicles are in the intelligent networking transportation system. The intelligent networking transportation system is a complex system with multiple elements, and its components are shown in Figure 1. With the development of autonomous driving technology, the research on the lane-changing decision making of autonomous vehicles has gradually become a hot spot. Some researchers have explored the lane-changing decision making of autonomous vehicles in different scenes. Wang et al. [27] determined the safe lane-changing threshold of autonomous vehicles under normal conditions. Smirnov et al. [28] studied the lane-changing decision making of autonomous vehicles at congested urban intersections. Gu et al. [29] explored the lane-changing decision-making process of autonomous vehicles in straight lanes or curved lanes on expressways. Gao et al. [30] analyzed the lane-changing decision making of autonomous vehicles in a mixed-traffic environment. Li et al. [31] proposed risk-aware lane-changing decision making for autonomous vehicles on normal roads. Wu et al. [32] studied the lane-changing decision making of autonomous vehicles on a straight and level road. An et al. [33] explored the lane-changing decision-making process of autonomous vehicles on a straight road under stable steering.

In summary, the previous studies on lane-changing decision-making behavior partly focus on the fixed lane-changing gap around the lane-changing vehicles, and the dynamic factors in the lane-changing process are less considered, which reduces the lane-changing rationality. To overcome the limitations of the previous studies, we make some innovations and improvements. The lane-changing behavior of vehicles is a kind of microscopic driving behavior with great randomness. Therefore, considering the system similarity, we compare microscopic vehicles to molecules and establish a molecular-dynamics lane-changing model of autonomous vehicles based on molecular-dynamics theory and the dynamic influencing factors. We explore the relationship between the lane-changing initial time and lane-changing completed time. We analyze the dynamic influencing factors of lane changing. We study the lane-changing characteristics of autonomous vehicles from the perspective of molecular dynamics, and we explore the impact of the lane-changing behavior on the macro traffic flow [34], so as to ensure the stable and efficient operation of autonomous vehicles.

The rest of the paper is organized as follows. Section 2 presents the environmental perception before lane-changing decision making, lane-changing behavior, and system similarity in terms of lane changing. Section 3 establishes the molecular-dynamics lane-changing model of autonomous vehicles. Section 4 analyzes the experimental environment and data. Section 5 evaluates the performance of the molecular-dynamics lane-changing model through experimental comparison. Section 6 concludes the paper.

## 2. Problem Formulation and Process Construction

### 2.1. Environmental Perception before Lane-Changing Decision Making

**Figure 2:**
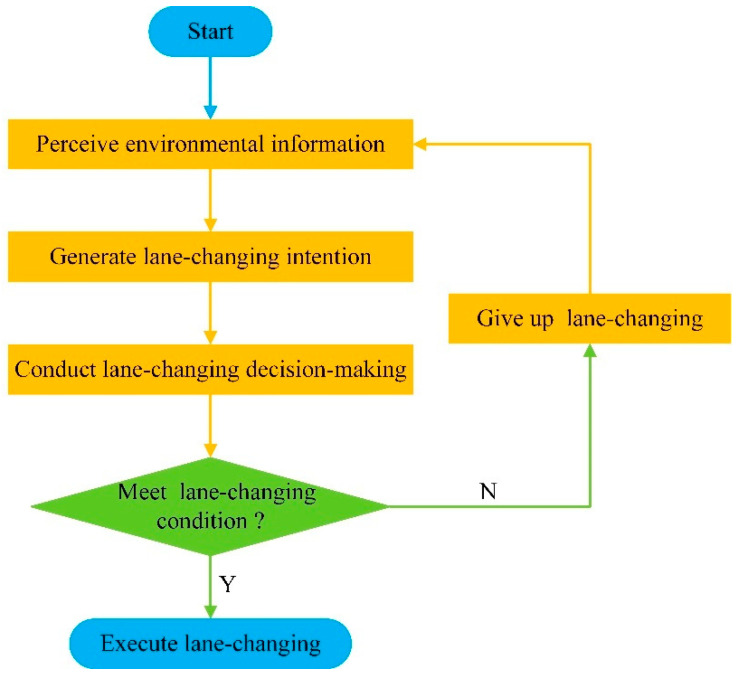
Process of lane-changing decision making.

The environmental-perception system is an essential part of the autonomous driving system. Its main task is to provide the basis for decision making. Autonomous vehicles need to perceive environmental information before decision making. The environmental perception will have an important impact on the lane-changing decision making. Achieving appropriate environmental information can improve the rationality of the lane-changing decision making. The process of lane-changing decision making is shown in Figure 2.

There are two kinds of environmental information. One is the vehicle’s own information, including the speed, acceleration, position, and steering angle. The other is the vehicle’s surrounding information, including the weather conditions, surrounding pedestrians or vehicles, traffic lights, buildings, and traffic signs.

The environmental-perception technology of autonomous driving has been developed. Tsukuba Engineering Research Laboratory (1977), in Japan, developed the first autonomous vehicle, which sensed the front marks based on the camera and navigated the information [35]. German military scientific research institutions cooperated with Mercedes Benz (1987) to develop autonomous vehicles, which used cameras and computer-image-processing systems to recognize roads, improving the environmental perception [36]. Stanford University and Volkswagen (2006) used radar and measurement units to connect a satellite system, perceiving the environments of vehicles [37]. Based on environmental perception, Zhejiang University (2012) made autonomous vehicles that have the functions of tracking roads, avoiding obstacles, and fork-road selection [38]. Google (2014) configured a variety of sensors on autonomous vehicles, such as laser radars, millimeter-wave radars, and infrared cameras, which achieved rich sensing functions [39]. The mainstream perception technologies include visual perception, laser perception, and microwave perception. Visual perception is based on image information collected by a camera, using the visual-correlation algorithm to process and recognize the environment information. Laser perception is based on point-cloud data collected by laser radar, through filtering, clustering, and other technologies, to perceive the environment. Microwave perception is based on the distance information collected by microwave radar, using the distance-correlation algorithm to process and recognize the environment information. A comparison of several perception technologies is shown in Table 1 [40].

**Figure 3:**
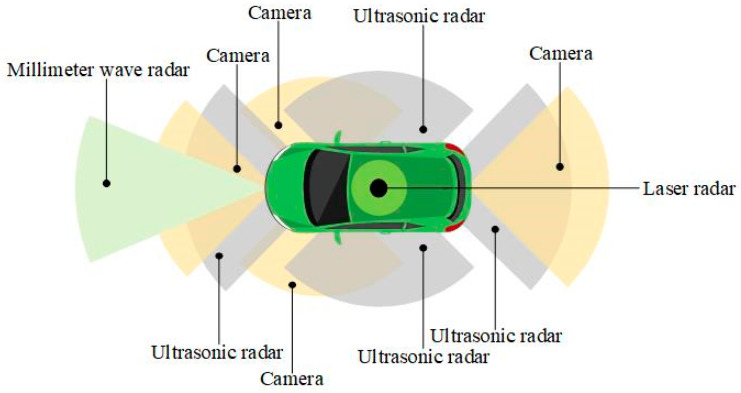
Types and layout of sensors on autonomous vehicles.

The sensors used in the environmental-perception systems of autonomous vehicles mainly include an on-board camera, laser radar, millimeter-wave radar, and ultrasonic radar. The camera is a commonly used sensor that can provide high-resolution images. Moreover, the camera processes the image and converts it into digital signals through the internal photosensitive components and control components so as to perceive the environment around the vehicles. Its environmental perception can be detailed to color and texture [41]. Laser radar uses remote-sensing technology, which generates 3D scenes in the form of point clouds when scanning the surrounding environment, and it analyzes the distance between the pulse transmission and reception. It has accurate 3D perception [42]. Millimeter-wave radar radiates the electromagnetic wave in the designated area and receives the reflected wave of the target, providing distance information for further signal processing. It uses the Doppler effect to calculate the speeds and positions of obstacles and finally provide reliable obstacle perception. It has a fast response speed and can provide effective environmental information in relatively complex weather [43]. Ultrasonic radar converts ultrasonic signals into other signals. It has strong penetration. It can help autonomous vehicles perceive the external environment, and it can promote the vehicles to make appropriate responses [44]. The types and layout of the sensors on autonomous vehicles are shown in Figure 3. Through the environmental perception of the sensors, autonomous vehicles can obtain traffic information and further make appropriate autonomous decisions. Finally, autonomous vehicles can run safely and smoothly.

In terms of the adaptability of autonomous vehicles, autonomous vehicles at this stage cannot fully adapt to actual complex traffic scenes. Therefore, the environmental-perception ability of autonomous vehicles needs to be further improved. With the improvement in autonomous driving technology, autonomous vehicles have higher requirements for environmental perception [45]. Different sensors have different advantages. Sensor-fusion technology is the development trend of the environmental-perception system. Integrating the information obtained by different sensors and analyzing it through computer technology to eliminate the redundancy and contradiction between the information will help to improve the perception ability of autonomous vehicles. At the same time, it will also promote autonomous vehicles to make fast and correct autonomous decisions.

### 2.2. Analysis of Lane-Changing Behavior

**Figure 4:**
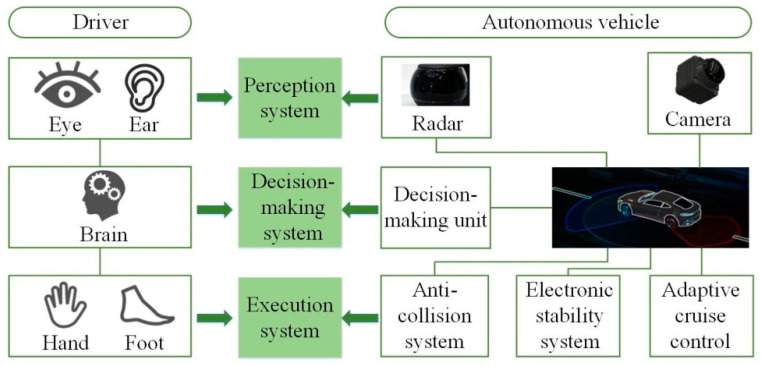
Components of an autonomous driving system.

In the intelligent networking environment, the autonomous driving system mainly includes three parts: environmental perception, decision planning, and execution control. These three parts have their own functions, and they cooperate with each other so as to ensure the safe and efficient driving of autonomous vehicles. The components of the autonomous driving system are shown in Figure 4. The perception system is equivalent to the driver’s eyes and ears. The vehicle realizes the perception of the environmental information through the perception system. The execution system is equivalent to the driver’s hands and feet. It can execute decision-making signals. In contrast, the decision-making system is similar to the brain of autonomous vehicles. By processing the environmental information obtained by the perception system, the decision-making system makes appropriate decisions for the driving of vehicles. The decision-making system embodies the intelligence of autonomous vehicles. Based on this, this paper studies the lane-changing decision making of autonomous vehicles.

When an autonomous vehicle is driving on the road, it may change from the current lane to the target lane through independent decision making [46]. An autonomous vehicle has no fatigue driving problem and a small delay, and so it can avoid traffic accidents caused by human factors and improve the driving safety and efficiency in the lane-changing process. In the study of driving behaviors, the car-following behavior and lane-changing behavior are two basic driving behaviors. Compared with the car-following behavior, the lane-changing behavior is more complex. Specifically, the lane-changing process includes the generation of the lane-changing intention, the formulation of the lane-changing decision making, and the implementation of the lane-changing process. In order to ensure smooth lane changing, time and space factors should be considered. On the one hand, for the time factor, autonomous vehicles need appropriate and continuous time to quickly change lanes. On the other hand, for the space factor, autonomous vehicles need a reasonable lane-changing distance to ensure the safety of the autonomous vehicles and surrounding vehicles.

**Figure 5:**
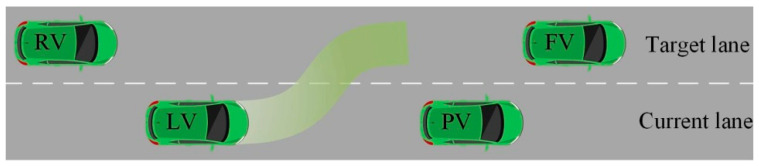
Free-lane-changing scene.

The lane-changing reasons mainly include increasing speed, avoiding obstacles, and road merging. According to the different lane-changing intentions, the lane-changing behaviors are divided into forced-lane-changing behavior and free-lane-changing behavior [47]. Forced lane changing is a necessary lane-changing behavior for vehicles to reach the destination. Forced lane changing has a certain impact on the completion of driving tasks. Vehicles will look for suitable opportunities to change lanes in the lane-changing area. In addition, the forced lane changing contains the latest lane-changing location, and the vehicle should change lanes before this location. If the lane changing is still not successful at this location, then parking and waiting may occur. Free-lane-changing behavior is an unnecessary lane-changing behavior to achieve the optimal driving state under the existing conditions. After changing lanes, the vehicle can increase the speed or obtain a more comfortable driving environment. Compared with forced lane changing, free lane changing has less impact on the completion of the driving tasks. Free lane changing has a certain degree of autonomy and non-necessity, and sometimes it will be affected by the traffic environment and will eventually give up lane changing. As shown in Figure 5, for the one-way double lane, there is an FV (front vehicle) and RV (rear vehicle) in the target lane. In addition, there is a PV (preceding vehicle) and LV (lane-changing vehicle) in the current lane [48]. They are all autonomous vehicles, and the LV is a lane-changing vehicle. The speed of the LV is constrained by the PV when driving, and the LV has the lane-changing intention to improve the driving environment. However, the LV has a high probability of collision with the RV during the lane changing, and so it finally gives up the lane changing. Therefore, the lane-changing process of the LV is free lane changing, which is unnecessary. The occurrence of the lane-changing behavior is the result of the interaction between the vehicles and the surrounding traffic environment. This paper explores the free-lane-changing behavior of autonomous vehicles.

### 2.3. Analysis of System Similarity of Lane Changing

By observing the traffic flow on the road from a high altitude, vehicles are similar to many tiny moving molecules. Molecules have certain similarities with vehicles. There are gaps between molecules, and vehicles maintain a certain distance from surrounding vehicles when driving. In addition, the smell of perfume indicates that there is a causal relationship between moving molecules and macroscopic phenomena. In the field of transportation, the lane changing of individual vehicles in the micro will sometimes cause a certain disturbance to the traffic flow in the target lane, and it will ultimately cause traffic congestion on the road in the macro.

**Figure 6:**
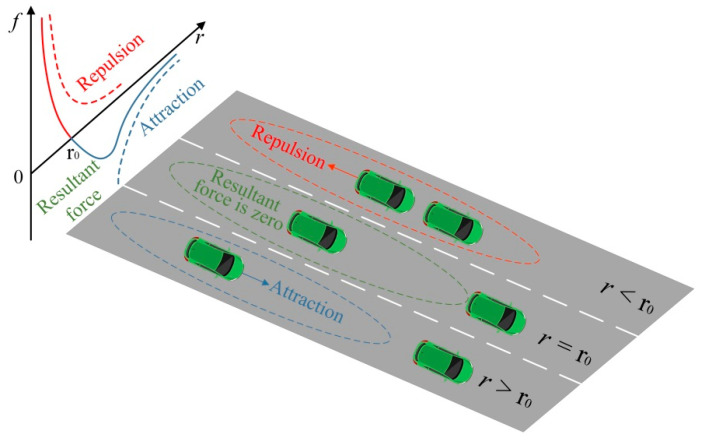
Interaction forces under different distances between vehicles.

For the molecular system, both attraction and repulsion exist between molecules, and they are affected by the molecular distance. The resultant force between molecules will also change according to the molecular distance. As shown in Figure 6, when the molecular distance (r) is less than the zero-point distance (r_0_), the resultant force appears as repulsion. When the molecular distance (r) is greater than the zero-point distance (r_0_), the resultant force appears as attraction. When the molecular distance (r) is equal to the zero-point distance (r_0_), the resultant force appears as zero [49]. The zero-point distance is also called the equilibrium distance. The equilibrium distance is not too large or too small. Under a certain interaction, the molecular distance will try to keep around the equilibrium distance, and it will eventually make the molecules tend to an equilibrium state. For the traffic system, the distance between vehicles is similar to the molecular distance. When the distance between vehicles is too small, the vehicles will actively increase the distance so as to ensure the safety of the vehicles. This situation is similar to a repulsive force between vehicles, which makes the vehicles move away from each other. When the distance between vehicles is too large, the vehicles will actively reduce the distance, thereby improving the driving efficiency and road utilization. This situation is similar to an attractive force between vehicles, which makes the vehicles move towards each other. When the vehicle is running, it always tries to maintain an appropriate equilibrium distance from the surrounding vehicles. That is to say, the vehicle tends to maintain a dynamic demand safety distance, which makes the vehicle neither lag nor follow closely, and it thus drives safely and efficiently.

**Figure 7:**
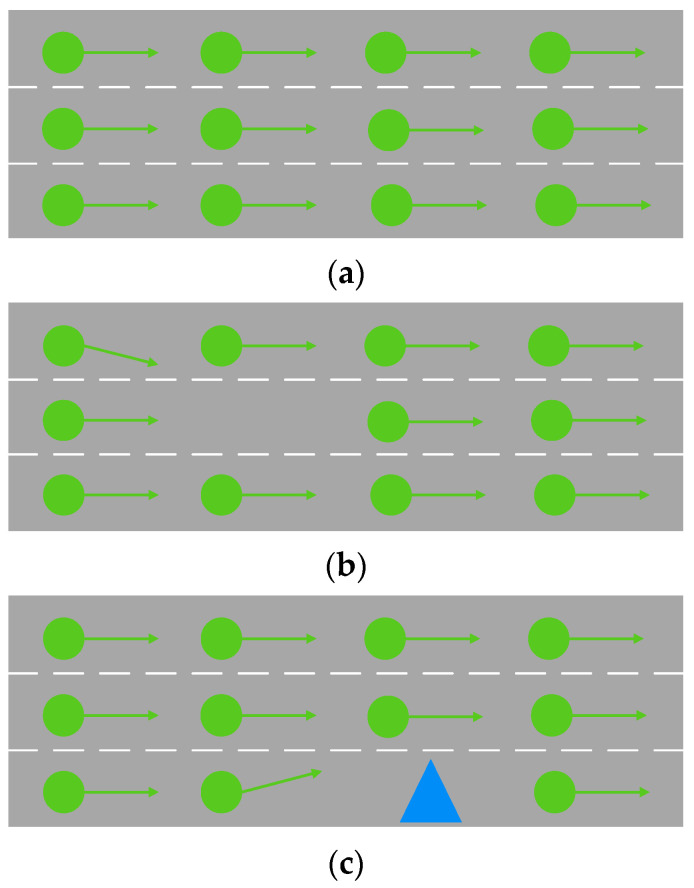
General molecular-motion states: (**a**) molecular tight-motion state; (**b**) molecular large-gap-motion state; (**c**) molecular hindered-motion state.

The following situations may occur for moving molecules, as shown in Figure 7. The motion of molecules is similar to that of vehicles. As shown in Figure 7a, when the molecules move tightly, the number of molecules in a region is large, and the motion speed is fast. This situation is similar to a large traffic flow and high vehicular operation efficiency on the road. As shown in Figure 7b, when there is a large gap in the molecular motion, some molecules will move to the large gap, and finally, the molecular distance will maintain a balanced state. This situation is similar to when there is a large gap in the target lane and the vehicle in the current lane changes lanes to improve the driving environment. As shown in Figure 7c, when the motion of molecules is hindered, the molecules will change the direction of motion. This situation is similar to a vehicle encountering an obstacle to create forced lane changing. The blue triangle represents an obstacle in Figure 7c. The traffic environment around the vehicle will affect the vehicle to produce some driving behaviors. At the same time, the driving behavior of the vehicles will also have an impact on the surrounding traffic environment so that the traffic environment will reach a new equilibrium state.

The complexity of macroscopic traffic flows is mainly due to the interaction behavior of microscopic vehicles. The lane changing is the behavior expression of the dynamic interaction between vehicles. The force causes a change in the motion state. In fact, the molecules move under the resultant force of the surrounding molecules, and the vehicles with lane-changing intentions will change lanes under the influence of the surrounding vehicles. Specifically, the vehicles in the target lane provide better speed conditions and appropriate lane-changing space, which will form the attraction effect on the lane-changing vehicles, and finally make the vehicles change lanes smoothly. Based on the molecular-dynamics analysis of lane-changing behavior, vehicles on the road are regarded as microscopic molecules. At the microscopic level, this paper scientifically shows the lane-changing conditions and lane-changing needs of vehicles so as to form a reasonable lane-changing decision-making mechanism.

## 3. Methodology

**Figure 8:**
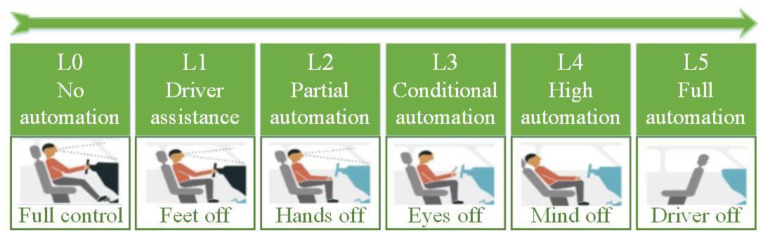
Levels of autonomous vehicles.

The lane-changing process of vehicles is generally complex, and so the lane-changing process of autonomous vehicles is modeled based on molecular dynamics from the microscopic perspective. Autonomous vehicles are usually classified by level. The higher the autonomous driving level, the higher the degree of the intelligence and automation [50]. As shown in Figure 8, according to the classification standard of the Society of Automotive Engineers (SAE), autonomous vehicles are divided into six levels, ranging from L0 to L5. L0 indicates that the vehicle is fully driven by the human driver. L1 indicates that the vehicle provides driving for one of the steering wheel, acceleration, or deceleration. Moreover, the human driver is responsible for the rest of the driving actions. L2 indicates that the vehicle provides driving for multiple operations in the steering wheel, acceleration, and deceleration. Moreover, the human driver is responsible for the rest of the driving actions. L3 indicates that the vehicle has completed most of the driving operations, but the human driver needs to pay attention in case of an emergency. L4 indicates that the vehicle has completed all the driving operations, and the human driver does not need to maintain attention, but the road and environmental conditions are limited. L5 means that the vehicle has completed all the driving operations, and the human driver does not need to pay attention. In addition, it is applicable to all scenes.

Autonomous vehicles have a certain interactivity. Combined with the development stage of autonomous driving, this paper describes autonomous vehicles as follows so as to better model the lane-changing decision-making behavior of autonomous vehicles:

(1) Autonomous vehicles have a high degree of automation and can operate autonomously;
(2) Autonomous vehicles can obtain information, such as the position and speed, in real time, and can conduct communication between vehicles;
(3) Autonomous vehicles are unified standard cars.

### 3.1. The Generation of the Lane-Changing Intention

The lane-changing process includes the generation of the lane-changing intention, the formulation of the lane-changing decision making, and the implementation of the lane-changing process. Autonomous vehicles need to generate the lane-changing intention before making the autonomous lane-changing decision. Generally, the speed factor is very important for the generation of the lane-changing intention. Specifically, in the free-lane-changing scene, the lane-changing intention of the autonomous vehicle is mainly affected by the speed of the front vehicle in the target lane and the preceding vehicle in the current lane [51]. The autonomous vehicle can objectively quantify its lane-changing intention by acquiring the speed information, as shown in Formula (1):

(1) $$k=\frac{v_{f}}{v_{p}}$$

In Formula (1), k is the lane-changing intention of the LV; $v_{f}$ represents the speed of the FV; $v_{p}$ represents the speed of the PV. The lane-changing intention of the LV is mainly determined by the ratio of the speed of the FV to the speed of the PV, and it is finally expressed by the value of k. When k>1, the target lane has better speed conditions than the current lane, and the autonomous vehicle has the lane-changing intention. The autonomous vehicle obtains speed benefits and improves the driving environment by changing lanes to complete the driving task efficiently. When k<1, the current lane still has good speed conditions. The autonomous vehicle has no intention to change lanes, and it will not change lanes in the end.

### 3.2. Molecular-Dynamics Lane-Changing Model

**Figure 9:**
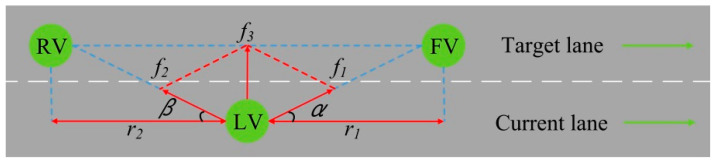
Force analysis of lane-changing vehicle molecule.

After generating the lane-changing intention, the autonomous vehicle needs to judge whether the surrounding environment is suitable for changing lanes safely and successfully. Generally speaking, the front vehicle and rear vehicle in the target lane have a great impact on the lane-changing process. As shown in Figure 9, the vehicles are compared to molecules, and the vehicle molecules are running along the centers of the lanes. Based on this, the force analysis of the lane-changing vehicle molecule is carried out so as to lay a foundation for the lane-changing decision making. $f_{1}$ is the force exerted by vehicle molecule FV on vehicle molecule LV. $f_{2}$ is the force exerted by vehicle molecule RV on vehicle molecule LV. $f_{3}$ is the resultant force received by vehicle molecule LV. α represents the horizontal angle for the connecting line of vehicle molecule LV and vehicle molecule FV. β represents the horizontal angle for the connecting line of vehicle molecule LV and vehicle molecule RV. $r_{1}$ represents the longitudinal distance between vehicle molecule LV and vehicle molecule FV. $r_{2}$ represents the longitudinal distance between vehicle molecule LV and vehicle molecule RV.

**Figure 10:**
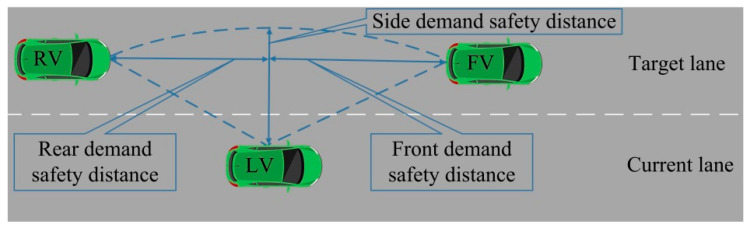
Demand safety distance of lane-changing vehicle.

In terms of the distance, the lane-changing vehicle needs the proper lane-changing distance to ensure the safety of the lane-changing process. Both the lateral distance and longitudinal distance should meet the lane-changing requirements. As shown in Figure 10, the lateral distance can generally be satisfied. However, the longitudinal distance is affected by the front and rear vehicles in the target lane, and it is unknown to some extent. Therefore, this paper focuses on the lane-change distance provided by the front and rear vehicles in the target lane.

By analyzing the positional relationship between the LV and FV at different stages during the lane-changing process, the following formula can be obtained:

(2) $$S_{1}\left(T\right)=S_{1}\left(0\right)+\left[x_{f}\left(T\right)-x_{f}\left(0\right)\right]-\left[x_{l}\left(T\right)-x_{l}\left(0\right)\right]$$

**Figure 11:**
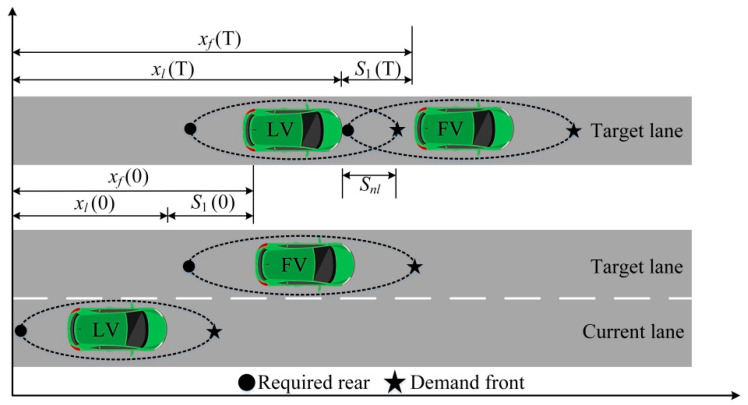
Positional relationships between LV and FV.

In Formula (2), $x_{f}(0)$ is the longitudinal position of the FV at the lane-changing initial time; $x_{f}(T)$ is the longitudinal position of the FV at the lane-changing completed time; $x_{l}(0)$ is the longitudinal position of the LV at the lane-changing initial time; $x_{l}(T)$ is the longitudinal position of the LV at the lane-changing completed time; $S_{1}(0)$ is the longitudinal distance between the LV and FV at the lane-changing initial time; $S_{1}(T)$ is the longitudinal distance between the LV and FV at the lane-changing completed time. The positional relationships between the LV and FV are shown in Figure 11.

There is a certain functional relationship between the headway and speed during the driving process of autonomous vehicles [52], and Formula (3) can be obtained through conversion:

(3) $$S_{n}=\eta v+\gamma v^{2}$$

In Formula (3), $S_{n}$ represents the demand safety distance of the vehicle; v is the speed; η represents the autonomous reaction time (autonomous vehicles are generally 0.1 s [53]); γ is one-half of the reciprocal of the maximum deceleration for the vehicle, and it is generally 0.07 m/s^2^ [54].

**Figure 12:**
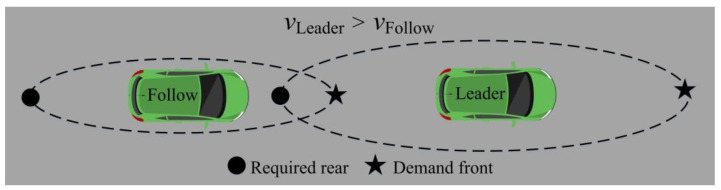
The demand safety distance of vehicles at different speeds.

From Formula (3), it can be seen that the demand safety distance of vehicles increases with the increase in the speed. On the contrary, the demand safety distance also decreases with the decrease in the speed. This phenomenon is similar to the “thermal expansion and cold contraction” that is caused by molecular motion. When the vehicle is running at a high speed, it is similar to the molecule in a high-temperature state. The vehicle needs to increase the distance between vehicles to ensure the driving safety. When the vehicle is running at a low speed, it is similar to the molecule in a low-temperature state. The vehicle can shorten the distance between vehicles to improve the driving efficiency and road utilization on the premise of ensuring the safety. At the same time, the dynamic changes in the distance with the speed also conform to reality. The demand safety distance of vehicles at different speeds is shown in Figure 12.

On the basis of the demand safety distance, and in combination with the actual distance between vehicles, the saturation coefficient of the demand distance can be further obtained, as shown in Formula (4):

(4) $$C_{n}=\frac{S_{n}}{L}$$

In Formula (4), $C_{n}$ represents the saturation coefficient of the demand distance, and L represents the actual distance between the vehicles.

The saturation coefficient of the demand distance can reflect some shortcomings of vehicles in operation. At the same time, the vehicle can improve problems to ensure safe and efficient driving. When $C_{n}<1$, the “demand front” of the rear vehicle does not touch the front vehicle, and so there is no risk of collision. However, from the perspective of lane utilization and traffic operation efficiency, the driving speed of the rear vehicle can be increased to make $C_{n}=1$. That is to say, the rear vehicle can make the “demand front” touch the rear boundary of the front vehicle to drive efficiently. When $C_{n}>1$, the “demand front” of the rear vehicle exceeds the rear boundary of the front vehicle. At this time, the rear vehicle is in danger of collision. Therefore, the rear vehicle should reduce the driving speed to make $C_{n}=1$. That is to say, the rear vehicle can make the “demand front” touch the rear boundary of the front vehicle to drive safely.

From the above analysis, it can be seen that if the LV can drive safely behind the FV after lane changing, then it is necessary to meet the saturation coefficient of the demand distance ($C_{nl}\leq 1$) for the lane-changing vehicle (LV). Formula (5) can be obtained by applying $C_{nl}\leq 1$ to Formulas (2) and (4). In addition, while considering the safety, the driving efficiency of vehicles should also be considered. Therefore, based on Formula (5), the initial expected safety distance between the LV and FV can be obtained, as shown in Formula (6):

(5) $$S_{1}\left(0\right)\geq S_{nl}+\left[x_{l}\left(T\right)-x_{l}\left(0\right)\right]-\left[x_{f}\left(T\right)-x_{f}\left(0\right)\right]$$

(6) $$S_{E}\left(l,f\right)=S_{nl}+\int_{0}^{t}\int_{0}^{\lambda }\left[a_{l}\left(\tau \right)-a_{f}\left(\tau \right)\right]d\tau d\lambda +\left[v_{l}\left(0\right)-v_{f}\left(0\right)\right]t$$

By analyzing Formulas (5) and (6), it can be seen that the initial expected safety distance ($S_{E}\left(l,f\right)$) is mainly composed of the demand safety distance ($S_{nl}$) of the lane-changing vehicle (LV) and the minimum lane-changing safety distance ($\left[x_{l}\left(T\right)-x_{l}\left(0\right)\right]-\left[x_{f}\left(T\right)-x_{f}\left(0\right)\right]$). In addition, the initial expected safety distance ($S_{E}\left(l,f\right)$) is a dynamic changeable distance, which is mainly affected by the speeds, accelerations, and lane-changing times of the LV and FV.

**Figure 13:**
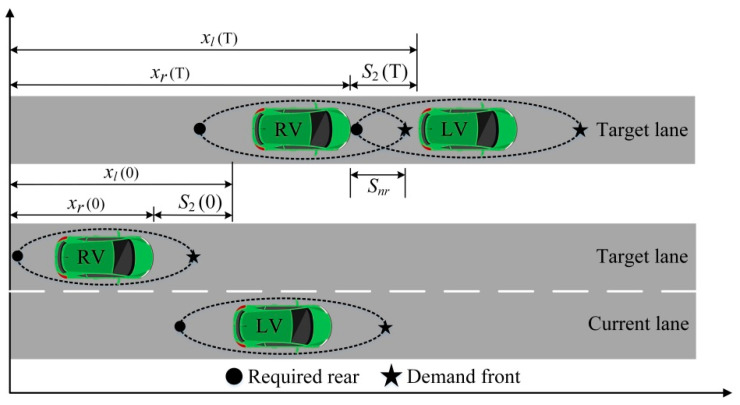
Positional relationships between LV and RV.

The front and rear vehicles in the target lane have an impact on the lane-changing decision making of the LV. Therefore, in addition to considering the positional relationship with the FV, the LV should also analyze the positional relationship with the RV. The positional relationships between the LV and RV at different stages are shown in Figure 13.

Based on the analysis process of the initial expected safety distance between the LV and FV, the similarity analysis of the lane-changing process can be carried out to derive the initial expected safety distance between the LV and RV, as shown in Formula (7):

(7) $$S_{E}\left(l,r\right)=S_{nr}+\int_{0}^{t}\int_{0}^{\lambda }\left[a_{r}\left(\tau \right)-a_{l}\left(\tau \right)\right]d\tau d\lambda +\left[v_{r}\left(0\right)-v_{l}\left(0\right)\right]t$$

On the basis of the initial expected safety distance between the LV and FV, combined with the interaction potential in molecular dynamics [55], the interaction potential of vehicle molecule LV and vehicle molecule FV can be obtained, as shown in Formula (8), where ε represents the depth of the potential well. By differentiating $\varphi_{1}(l)$ with respect to $S_{1}(0)$, the force ($f_{1}\left(l\right)$) exerted by the FV on the LV at the lane-changing initial time can be obtained, as shown in Formula (9). Using the relationship between the force and acceleration, the acceleration ($a_{1}\left(l\right)$) given by the FV to the lane-changing vehicle (LV) can be finally obtained, as shown in Formula (10), where m represents the mass of the LV:

(8) $$\varphi_{1}(l)=4\varepsilon \left[{\left(\frac{S_{E}(l,f)}{S_{1}(0)}\right)}^{12}-{\left(\frac{S_{E}(l,f)}{S_{1}(0)}\right)}^{6}\right]$$

(9) $$f_{1}\left(l\right)=-24\varepsilon \left[2\frac{S_{E}{\left(l,f\right)}^{6}}{S_{1}{\left(0\right)}^{7}}-\frac{1}{S_{1}\left(0\right)}\right]{\left(\frac{S_{E}\left(l,f\right)}{S_{1}\left(0\right)}\right)}^{6}$$

(10) $$a_{1}\left(l\right)=\frac{-24\varepsilon }{m}\left[2\frac{S_{E}{\left(l,f\right)}^{6}}{S_{1}{\left(0\right)}^{7}}-\frac{1}{S_{1}\left(0\right)}\right]{\left(\frac{S_{E}\left(l,f\right)}{S_{1}\left(0\right)}\right)}^{6}$$

By analogy with the reasoning process of $a_{1}\left(l\right)$, the acceleration ($a_{2}\left(l\right)$) given by the RV to the lane-changing vehicle (LV) can be obtained, as shown in Formula (11):

(11) $$a_{2}\left(l\right)=\frac{-24\varepsilon }{m}\left[2\frac{S_{E}{\left(l,r\right)}^{6}}{S_{2}{\left(0\right)}^{7}}-\frac{1}{S_{2}\left(0\right)}\right]{\left(\frac{S_{E}\left(l,r\right)}{S_{2}\left(0\right)}\right)}^{6}$$

Let $\mu =\frac{-24\varepsilon }{m}$ and $G_{1}=\frac{S_{E}(l,f)}{S_{1}(0)}$, and then Formula (10) can be converted into Formula (12):

(12) $$a_{1}\left(l\right)=\mu \left[\frac{2{G_{1}}^{6}-1}{S_{1}\left(0\right)}\right]{G_{1}}^{6}$$

At the same time, let $G_{2}=\frac{S_{E}(l,r)}{S_{2}(0)}$, and then Formula (11) can be converted into Formula (13):

(13) $$a_{2}\left(l\right)=\mu \left[\frac{2{G_{2}}^{6}-1}{S_{2}\left(0\right)}\right]{G_{2}}^{6}$$

Combined with the analysis of the lane-changing vehicle molecule (LV) in Figure 9, the obtained accelerations can be decomposed and recombined to objectively derive the lateral acceleration ($a_{h}$) and longitudinal acceleration ($a_{z}$) of the LV, as shown in Formulas (14) and (15), respectively:

(14) $$a_{h}=a_{1}\left(l\right)\sin\alpha +a_{2}\left(l\right)\sin\beta$$

(15) $$a_{z}=a_{1}\left(l\right)\cos\alpha +a_{2}\left(l\right)\cos\beta$$

Taking Formulas (12) and (13) into Formulas (14) and (15), respectively, the final expressions of the lateral acceleration ($a_{h}$) and longitudinal acceleration ($a_{z}$) of the lane-changing vehicle (LV) can be obtained, as shown in Formulas (16) and (17), respectively:

(16) $$a_{h}=\mu \left[\frac{2{G_{1}}^{6}-1}{S_{1}\left(0\right)}\right]{G_{1}}^{6}\sin\alpha +\mu \left[\frac{2{G_{2}}^{6}-1}{S_{2}\left(0\right)}\right]{G_{2}}^{6}\sin\beta$$

(17) $$a_{z}=\mu \left[\frac{2{G_{1}}^{6}-1}{S_{1}\left(0\right)}\right]{G_{1}}^{6}\cos\alpha +\mu \left[\frac{2{G_{2}}^{6}-1}{S_{2}\left(0\right)}\right]{G_{2}}^{6}\cos\beta$$

When the resultant force exerted by the front and rear vehicle molecules in the target lane is attraction, the lane-changing vehicle molecule tends to enter the target lane. At this time, the vehicle can change lanes. When the resultant force exerted by the front and rear vehicle molecules in the target lane is repulsion, the lane-changing vehicle molecule tends to stay in the current lane. At this time, the vehicle does not change lanes. Because the force has a close relationship with the acceleration, the acceleration can be analyzed to conduct the lane-changing decision making. When $a_{h}>0$, the resultant force exerted by the front and rear vehicles in the target lane is attraction, and the lane-changing vehicle can change lanes by adjusting the speed. When $a_{h}\leq 0$, the resultant force exerted by the front and rear vehicles in the target lane is either repulsion or zero. Therefore, the vehicle should give up lane changing and drive in the current lane. To sum up, the autonomous vehicle makes the independent lane-changing decision based on the acceleration so as to ensure that the vehicle can smoothly implement the lane changing.

## 4. Results

### 4.1. Experimental Platform and Environment

In this study, lane-changing information was obtained, and a lane-changing experiment was carried out through the SUMO platform [56]. SUMO is an open-source and multimodal traffic experimental platform that can realize and evaluate traffic research. It has built-in lane-changing models and a set of tools for scene creation, which can reasonably conduct lane-changing experiments. SUMO contains a road-network editor, which can edit the connection relationships among lanes. At the same time, SUMO contains some application tools, which are shown in Table 2. In addition, the TraCI (Traffic Control Interface) within SUMO can track the vehicle and obtain its lane-changing information. Furthermore, SUMO can also run jointly with other simulation programs to meet specific requirements. In the intelligent networking environment, SUMO is gradually being applied to autonomous driving research, which also promotes the development of autonomous driving technology to a certain extent.

### 4.2. Analysis of Experimental Data

**Figure 14:**
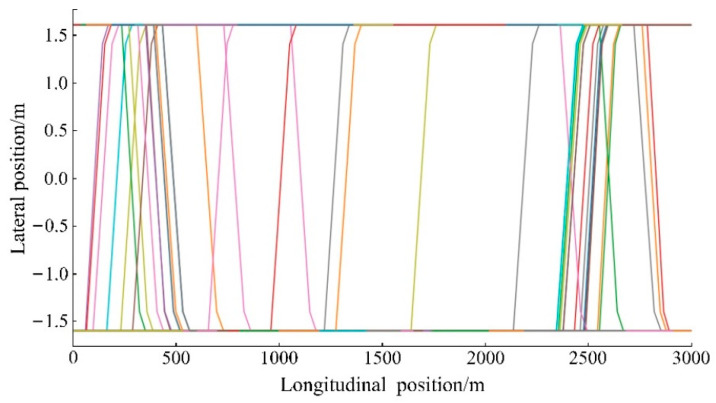
Trajectory information of vehicles.

The experimental environment was set as the autonomous driving environment. In addition, the experimental scene was set as a one-way two-lane road with a length of 3000 m and a speed limit of 120 km/h. First, in one experiment, the trajectory information of the vehicles within 300 s was extracted, and the result is shown in Figure 14. The operation of vehicles has a certain randomness. After sensing environmental information through sensors, autonomous vehicles will make decisions to meet the requirements according to the changeable traffic environment, and they will ultimately produce different driving behaviors. It can be seen from Figure 14 that the vehicles have different driving trajectories, and the lane-changing processes are also different. The longitudinal position of the vehicle always changes during the driving process. Moreover, the lateral position of the vehicle will also change during the lane-changing process. Because a one-way two-lane road was set in the experiment and the vehicle drove along the centers of the lanes, the lane-changing width of the vehicle was usually the width of a single lane. When the vehicle drives at a distance of 1500~2000 m, the traffic environment is relatively good, and the running speed of the vehicle is high. At this distance, the lane-changing intentions of the vehicles are not strong, and so the lane-changing trajectories are relatively few.

**Figure 15:**
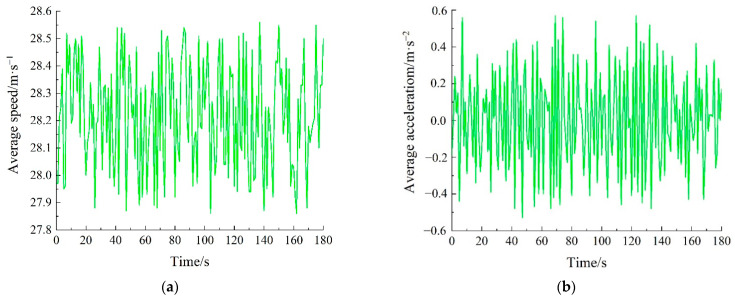
Operations of vehicles: (**a**) average speed; (**b**) average acceleration.

In order to study the overall operation of the vehicles, the average speed and average acceleration of the vehicles were analyzed, as shown in Figure 15. Figure 15a shows the average speed of the vehicles. The autonomous vehicles accelerated and decelerated autonomously according to the surrounding traffic environment. The vehicles drove on the highway and there were relatively few obstacles. Therefore, the vehicles ran stably, with small speed fluctuations, on the whole. Figure 15b shows the average acceleration of the vehicles. The acceleration of the vehicles shows a fluctuating state, and the variation range of acceleration is −0.6~0.6 m/s^2^, which means that the vehicles show relatively small fluctuations in speed. In short, the vehicles can drive in a stable state on the road.

**Figure 16:**
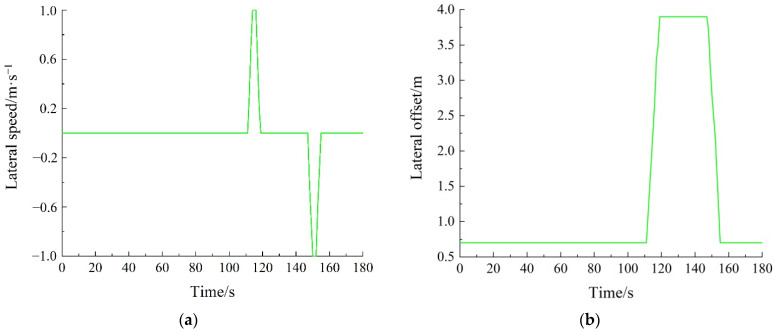
Information on lane-changing vehicle: (**a**) lateral speed of lane-changing vehicle; (**b**) lateral offset of lane-changing vehicle.

TraCI was used to track the lane-changing vehicle in the SUMO platform, and the relevant lane-changing information obtained is shown in Figure 16. Figure 16a shows the lateral speed of the lane-changing vehicle. When the vehicle drives along the center of the current lane, if the lateral speed of the vehicle does not change, then this indicates that the vehicle has not changed lanes. If the lateral speed of the vehicle changes, then this indicates that the vehicle has changed lanes. At the initial stage of being tracked, the lateral speed of the vehicle is zero, which means that the vehicle does not change in the lateral position. That is to say, the vehicle does not change lanes at the initial stage of being tracked. When the vehicle changes lanes, its lateral speed increases first, and then decreases. After reaching the target lane, the lateral speed of the vehicle becomes zero again. Figure 16b shows the lateral offset of the lane-changing vehicle. Specifically, it shows the offset of the right side of the lane-changing vehicle relative to the right side of the road. Furthermore, it shows the variation in the lateral position for the vehicle. In the driving process, the longitudinal offset of the vehicle is constantly increasing, and the lane-changing information of the vehicle cannot be directly observed based on the longitudinal offset. Therefore, the lateral offset of the vehicle is studied to obtain the lane-changing information. If the vehicle has been driving in the current lane, then the lateral offset of the vehicle remains unchanged. If the vehicle changes lanes, then the lateral offset of the vehicle changes. The experimental scene is a one-way two-lane road, and the vehicle drives along the center line of the lane. Therefore, the lateral offset of a lane change is the width of a single lane. In conclusion, the vehicle changes lanes twice during the tracked time. In addition, the lane-changing information expressed in Figure 16a,b is also consistent.

## 5. Discussion

In the SUMO platform, the lane-changing process of the vehicle can be observed, and the lane-changing information of the vehicle can be obtained. Based on the obtained lane-changing data, the parameters of the molecular-dynamics lane-changing model can be calibrated [57], which can make the molecular-dynamics lane-changing model practical. In addition, it is helpful for the vehicle to make the reasonable lane-changing decision. After the parameter calibration, the molecular-dynamics lane-changing model can be clarified so that its performance can be objectively analyzed and evaluated. The parameter calibration of the molecular-dynamics lane-changing model is shown in Table 3.

**Figure 17:**
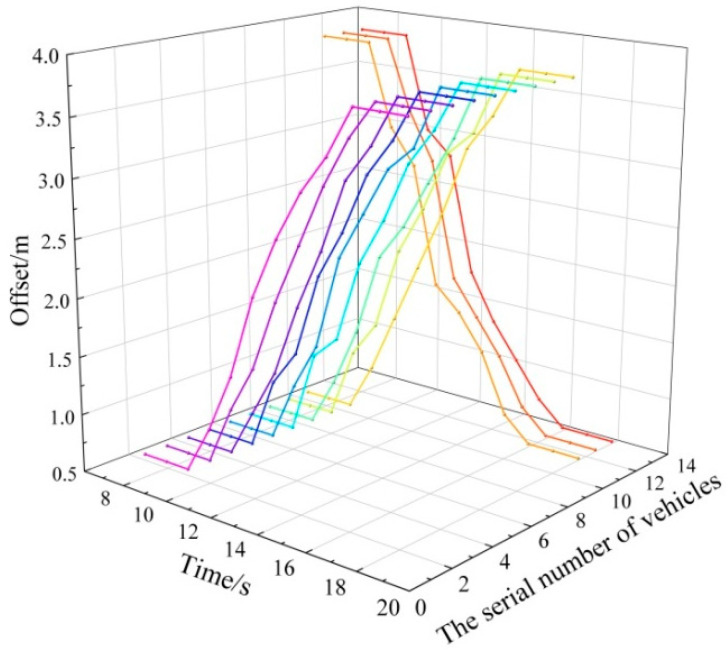
Lane-changing information of vehicles under molecular-dynamics lane-changing model.

The lane-changing information of vehicles under the molecular-dynamics lane-changing model was extracted to analyze the performance of the model. In the experimental scene, the width of a single lane was set to 3.2 m, and the width of a vehicle was set to 1.8 m. In addition, when the vehicle did not change lanes, it drove along the center lines of the lanes. Therefore, the fluctuation range of the offset of the right side of the vehicle relative to the right side of the road was 0.7~3.9 m. For the vehicles driving on one-way two-lane roads, the lane-changing information was extracted within the set time, and the results are shown in Figure 17. It can be seen from the figure that twelve vehicles implemented the lane-changing process. Along the traffic-flow direction, nine vehicles changed from the right lane to the left lane, and three vehicles changed from the left lane to the right lane. Compared with the left lane, the traffic flow in the right lane was larger, and the autonomous vehicles expected to achieve speed gains and improve the driving environment. Therefore, in contrast, more vehicles changed lanes from the right lane to the left lane, which also conforms to reality. Autonomous vehicles will make decisions to meet the requirements according to the changeable traffic environment, thus generating different driving behaviors to complete the driving tasks. In conclusion, the molecular-dynamics lane-changing model is reasonable and practical, and it also promotes the harmonious driving of autonomous vehicles on the road.

In the above experimental environment, the molecular-dynamics lane-changing model was compared with the SL2015 lane-changing model so as to more intuitively evaluate the performance of the molecular-dynamics lane-changing model. The SL2015 lane-changing model is a model within SUMO that can be used for sublane simulation, and it has a high resolution during the experiment. When the vehicle constantly moves between lanes, multiple steps are required to achieve the movement. In addition, the behaviors of the vehicle are affected by the attributes of the lane-changing model, and the vehicle maintains the distance based on a distance that is not too far. The width of the lanes affects the fidelity with regard to the acceptance of the lateral gaps, and it also determines the number of candidate movements that are evaluated during the lane changing. The SL2015 lane-changing model can simulate phenomena related to lateral vehicular dynamics, and its additional behavior layer can maintain safe lateral gaps. In addition, the vehicle can be positioned to the precise longitudinal and lateral positions by matching the specified coordinates. In short, the SL2015 lane-changing model can reasonably show the lane-changing behavior of vehicles. Table 4 shows the additional parameters supported by the SL2015 lane-changing model.

In addition, Table 5 shows the characteristic parameters of the vehicles under the SL2015 lane-changing model and molecular-dynamics lane-changing model.

**Figure 18:**
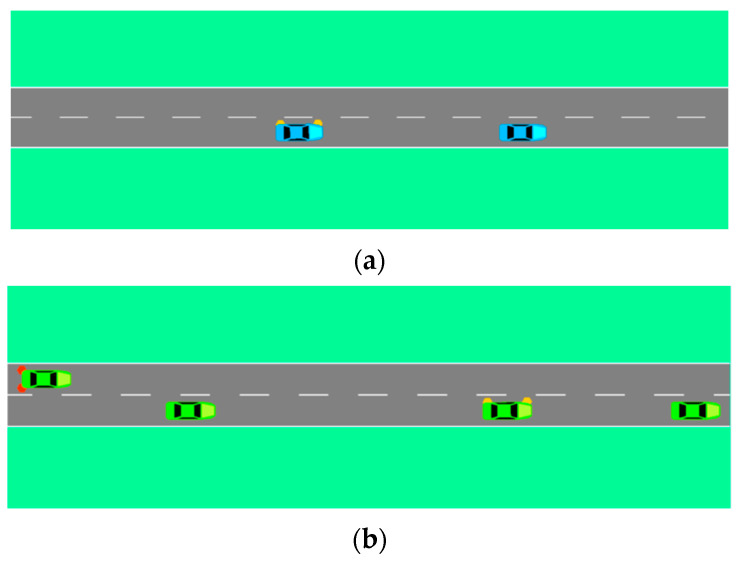
Operations of vehicles under two models: (**a**) operation of vehicles under SL2015 lane-changing model; (**b**) operation of vehicles under molecular-dynamics lane-changing model.

Figure 18 shows the operations of the vehicles under two models. When the vehicle is driving in the current lane, it will turn on the turn-signal light to implement the lane changing after it generates the lane-changing intention and makes the lane-changing decision. In addition, the rear vehicle in the target lane may sometimes brake and decelerate to ensure the safety of the vehicles after finding the lane-changing vehicle.

**Figure 19:**
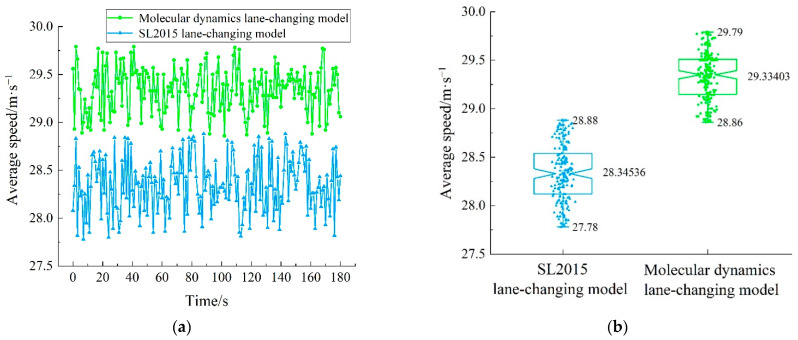
The average speeds of lane-changing vehicles under different models: (**a**) qualitative diagram of average speed; (**b**) quantitative diagram of average speed.

As shown in Figure 19, first, the performance of the molecular-dynamics lane-changing model was analyzed by comparing the two lane-changing models in terms of the average speed. Figure 19a shows that, compared with the SL2015 lane-changing model, the average speed of the vehicles under the molecular-dynamics lane-changing model was larger, and the fluctuation was smaller. On the basis of Figure 19a,b further objectively shows the increased amount of the average speed and decreased amount of the speed fluctuation under the molecular-dynamics lane-changing model. Figure 19b is a box–line diagram. The three data around each box are the maximum value, average value, and minimum value from top to bottom. Compared with the SL2015 lane-changing model, the average speed was increased by 3.49%, and the speed fluctuation was decreased by 15.45%, under the molecular-dynamics lane-changing model. In addition, there was no collision accident under the molecular-dynamics lane-changing model during the experiment. In conclusion, the molecular-dynamics lane-changing model established in this paper has better safety and efficiency. The molecular-dynamics lane-changing model enables the autonomous vehicle to consider the safety and efficiency when making the lane-changing decision, and to dynamically adjust the speed in the face of changeable traffic environments, which enables the control layer of the autonomous vehicle to implement lane changing more smoothly.

**Figure 20:**
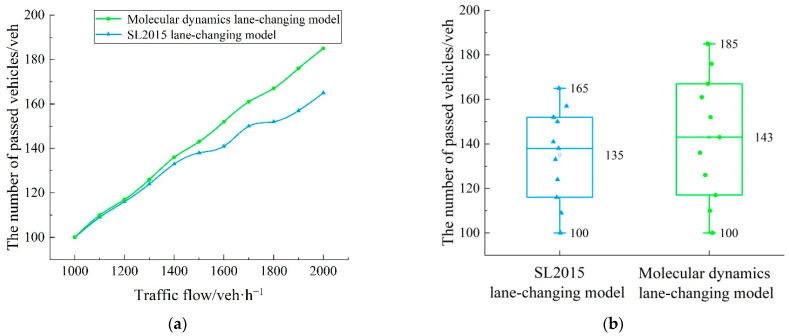
Numbers of passed vehicles under different models: (**a**) qualitative diagram of number of passed vehicles; (**b**) quantitative diagram of number of passed vehicles.

As shown in Figure 20, the performance of the molecular-dynamics lane-changing model was analyzed based on the number of passed vehicles. Figure 20a shows the change in the number of passed vehicles with the traffic flow. In the experimental scene, when the traffic flow was increasing, the number of passed vehicles under two models was increasing. However, there are differences in the specific change in the number of passed vehicles between the two models. Compared with the SL2015 lane-changing model, the number of passed vehicles under the molecular-dynamics lane-changing model is more, and this situation is more obvious when the traffic flow is more than 1500 veh/h. Figure 20b further intuitively shows the increased in the number of passed vehicles under the molecular-dynamics lane-changing model. Compared with the SL2015 lane-changing model, the number of passed vehicles under the molecular-dynamics lane-changing model increased by 5.93%. In conclusion, the molecular-dynamics lane-changing model has better road utilization and efficiency. For the development of traffic, the number of vehicles on the road is gradually increasing, which leads to an increasing demand of vehicles for road utilization. Therefore, the molecular-dynamics lane-changing model also has adaptability and practical applicability to the current and future traffic scenes.

**Figure 21:**
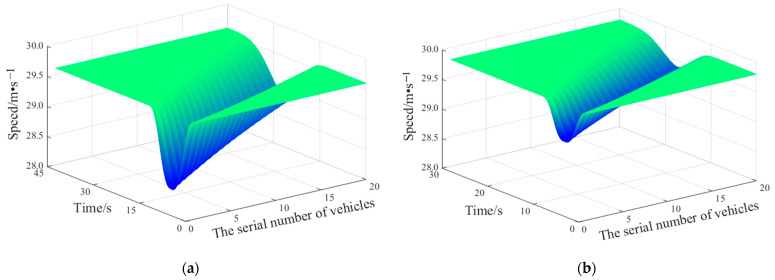
Disturbances of lane-changing vehicles under different models: (**a**) disturbance of lane-changing vehicles under SL2015 lane-changing model; (**b**) disturbance of lane-changing vehicles under molecular-dynamics lane-changing model.

The lane-changing behavior of lane-changing vehicles may cause certain disturbances to the traffic flow of the target lane. The smaller the disturbance of the lane-changing vehicles, the smaller the delay of the vehicles in the target lane. Finally, the vehicles can drive more efficiently. Therefore, the performance of the molecular-dynamics lane-changing model is analyzed based on the disturbance. A fleet of 20 vehicles is set in the rear of the target lane, and the vehicles in the fleet follow the same rules [58]. The lane-changing vehicle driving in the current lane seeks opportunities for lane changing under the lane-changing rules of the SL2015 lane-changing model and molecular-dynamics lane-changing model. As shown in Figure 21, after the vehicles in the fleet are disturbed by the lane-changing vehicle, the speed fluctuation will propagate backward, and the fluctuation degree will gradually decrease. This situation is also consistent with the actual scene. However, the disturbance of the lane-changing vehicle to the target traffic flow is different under the two lane-changing models. Under the molecular-dynamics lane-changing model, when the traffic environment of the target lane gives the attraction effect to the lane-changing vehicle of the current lane, the lateral acceleration of the lane-changing vehicle will be expressed as $a_{h}>0$, and the vehicle will change lanes. Compared with the SL2015 lane-changing model, the lane-changing vehicle under the molecular-dynamics lane-changing model has less disturbance to the traffic flow of the target lane. After being disturbed by the lane-changing vehicle under the molecular-dynamics lane-changing model, the traffic flow of the target lane can recover the previous driving state in a shorter time, and the speed fluctuation is smaller, which makes the traffic flow drive more stably and efficiently. In conclusion, the molecular-dynamics lane-changing model has better stability and efficiency. The lane-changing vehicle under the molecular-dynamics lane-changing model is good at selecting the lane-changing time and making friendly lane changing. In addition, the lane-changing vehicle creates less disturbance to the traffic flow, which promotes the harmonious driving of vehicles and alleviates traffic congestion to a certain extent.

## 6. Conclusions

Compared with the traditional lane-changing model, which focuses on the fixed critical gap, this paper explores the relationship between the lane-changing initial time and lane-changing completed time. In addition, the dynamic influencing factors of lane changing are studied. Based on the molecular-dynamics theory, the microscopic lane-changing behavior of vehicles is analyzed, and the molecular-dynamics lane-changing model of autonomous vehicles is established. Through reasoning analysis and simulation experiments, the following conclusions are obtained.

The lane-changing of autonomous vehicles has dynamics and interactivity. Through an analysis of system similarity, the vehicle is compared to the molecule. Applying molecular dynamics to the lane-changing scene in the autonomous driving environment can reasonably show the microscopic lane-changing behavior of vehicles.

On the basis of quantifying the lane-changing intention, the molecular-dynamics lane-changing model is established by analyzing the dynamic influencing factors in the lane-changing process, and by further introducing the interaction potential. While scientifically demonstrating the lane-changing-behavior characteristics of autonomous vehicles, it also promotes the safe and efficient lane changing of autonomous vehicles. The experimental results show that, compared with the SL2015 lane-changing model, the average speed of vehicles is increased by 3.49%, the speed fluctuation is reduced by 15.45%, and the number of passed vehicles is increased by 5.93% under the molecular-dynamics lane-changing model. In addition, under the molecular-dynamics lane-changing model, the disturbance of the lane-changing vehicle is smaller and there is no collision accident. Therefore, the molecular-dynamics lane-changing model has better safety, stability, and lane utilization. Moreover, this study is adapted to the development stage of autonomous vehicles, and it can provide a theoretical basis for the lane-changing research of autonomous vehicles to a certain extent.

The molecular-dynamics lane-changing model established in this paper is suitable for the traffic scene of the expressway. With the gradual popularization of autonomous vehicles, it is necessary to further study the lane-changing decision-making behavior of autonomous vehicles. On the one hand, more abundant lane-changing influencing factors will be comprehensively considered to make the lane-changing behavior of autonomous vehicles more reasonable. On the other hand, the molecular-dynamics lane-changing model will be improved so as to make the model suitable for more complex traffic scenes, and to promote the development of autonomous driving technology.

## Figures and Tables

**Table 1:** Comparison of perception technologies.

Perception Technology	Advantages	Disadvantages
Visual perception	Rich information	Susceptible to light and speed Low measurement accuracy of 3D information
	Good real-time performance	
	Low energy consumption	
Laser perception	Obtains 3D distance directly	Inability to perceive target information without distance difference in plane
	High measurement accuracy	
	Insensitive to light changes	
Microwave perception	Obtains 3D distance directly	Inability to perceive target information without distance difference in plane
	Good real-time performance	
	High measurement accuracy	

**Table 2:** Introduction to internal application tools of SUMO.

Name of Application Tool	Introduction
GUISIM	Application of graphic interface in microsimulation
NETCONVERT	Reads the road network in different formats and converts it to SUMO format
GETGEN	Generates abstract road networks for SUMO simulation
DUAROUTER	Calculates the shortest path
JTRROUTER	Calculates the path by using the intersection-turning ratio
DFROUTER	Calculates the path through the induction coil
OD2TRIPS	Obtains the path of a single vehicle
POLYCONVERT	Converts to visual content accepted by GUISIM

**Table 3:** Parameter calibration of molecular-dynamics lane-changing model.

Parameter	Value
*α*	0.0541
*β*	0.0506
*μ*	0.0056

**Table 4:** Additional parameters supported by SL2015 lane-changing model.

Additional Parameter	Range
lcSublane	[0–inf)
lcStrategic	[0–inf)
lcSpeedGain	[0–inf)
lcKeepRight	[0–inf)
lcCooperative	[0–1]
lcPushy	[0–1]
lcAssertive	[0–1)
lcImpatience	[−1–1]

**Table 5:** Characteristic parameters of vehicles.

Parameter	SL2015 Lane-Changing Model	Molecular-Dynamics Lane-Changing Model
Vehicle length (m)	4.8	4.8
Vehicle width (m)	1.8	1.8
Vehicle color	Deep sky blue	Lawn green
Maximum speed (m/s)	33.33	33.33

## Data Availability

Not applicable.
